# Three doses of BNT162b2 vaccine confer neutralising antibody capacity against the SARS-CoV-2 Omicron variant

**DOI:** 10.1038/s41541-022-00459-z

**Published:** 2022-03-08

**Authors:** Kevin K. Ariën, Leo Heyndrickx, Johan Michiels, Katleen Vereecken, Kurt Van Lent, Sandra Coppens, Betty Willems, Pieter Pannus, Geert A. Martens, Marjan Van Esbroeck, Maria E. Goossens, Arnaud Marchant, Koen Bartholomeeusen, Isabelle Desombere

**Affiliations:** 1 Virology Unit, Department of Biomedical Sciences, Institute of Tropical Medicine Antwerp, Antwerp, Belgium; 2 Department of Biomedical Sciences, University of Antwerp, Antwerp, Belgium; 3 Department of Clinical Sciences, Institute of Tropical Medicine Antwerp, Antwerp, Belgium; 4 SD Epidemiology and Public Health, Sciensano, Brussels, Belgium; 5 Department of Laboratory Medicine, AZ Delta General Hospital, Roeselare, Belgium; 6 Institute for Medical Immunology and ULB Center for Research in Immunology (U-CRI), Université libre de Bruxelles (ULB), Gosselies, Belgium; 7 Immune response, SD Infectious Diseases in Humans, Sciensano, Brussels, Belgium

**Keywords:** Viral infection, Medical research

## Abstract

We report the levels of neutralising antibodies against Wuhan, Delta and Omicron variants in unimmunized infected (group 1), immunised and boosted (group 2) and infected immunised and boosted (group 3) adult individuals. Our observations support the rapid administration of a booster vaccine dose to prevent infection and disease caused by Omicron.

Researchers in Botswana and South Africa identified a new and heavily mutated SARS-CoV-2 variant (B.1.1.529, Omicron) in late November 2021, with 30 amino acid mutations in the Spike protein that are distinct compared to other variants of concern (VOC) Alpha, Beta and Delta^1^. Omicron is characterised by fast-spreading in previously vaccinated populations, suggesting Omicron’s ability to evade vaccine-induced immunity^2^ and therapeutic monoclonal antibody therapy^3^. Several recent studies have indeed confirmed a substantial reduction in neutralising antibody activity against Omicron in small-scaled studies including previously-infected individuals, fully vaccinated individuals, recipients of third booster doses of BNT162b2 or mRNA-1273 and individuals with hybrid immunity (infection followed by vaccination)^4–9^. The common trend from these first laboratory-based assessments is that the potency to neutralise Omicron is reduced by ~40-fold (20–200-fold depending on the study) compared to the original Wuhan D614G virus.

Here, we have assessed the levels of neutralising antibodies (nAb) against the original lineage B (Wuhan-Hu-1), Delta (B.1.617.2) and Omicron (B.1.1.529) VOC of 30 sera collected from individuals infected with SARS-CoV-2 prior to vaccination (Group 1), from infection-naïve individuals after three doses of BNT162b2 (Group 2) and from previously-infected individuals after three doses of BNT162b2 (Group 3). Ethics approval and informed consent was obtained from all participants. Group 2 and 3 samples were collected as part of the PICOV study (Clinicaltrials.gov NCT04527614).

We tested three representative patient groups for neutralising antibody capacity against lineage B, Delta and Omicron variants of SARS-CoV-2 in a whole-virus neutralisation assay (Fig. 1). Group 1 sera (*n* = 10) were obtained from COVID-19 patients hospitalised with severe infection, requiring ICU and ventilation. Half of these patients (5/10) had multiple comorbidities. All patients were infected between 24 Feb 2020 and 27 March 2020 when lineage B (Wuhan-Hu-1) was the only variant circulating and when vaccines were not yet available. Samples tested were collected with a median time after the onset of symptoms of 25 days [range 13–46]. Group 2 sera (*n* = 10) were obtained from individuals without a documented previous SARS-CoV-2 infection and 28 days after the third dose of BNT162b2. All individuals were vaccinated with a 21-day interval between doses 1 and 2 and received third dose 7 months (median 211 days [207–219]) after dose 2. Group 3 sera (*n* = 10) were obtained from individuals with hybrid immunity, i.e. individuals who have had a previous infection with lineage B (Wuhan-Hu-1) between 24 March 2020 and 11 June 2020, followed by three doses of the BNT162b2 vaccine. Dose 1 and dose 2 were given 21-days apart and the third dose was administered with a median time interval of 8 months (median 261 days [218–290]) after dose 2. Samples tested for group 3 were collected with a median time after third vaccine dose of 14 days [range 10–82]. All participants were of European origin, with a mean age of 71 years [range 53–84; 40% females], 51 years [range 28–88, 70% females] and 67 years [range 22–95, 50% females] for groups 1, 2 and 3, respectively. Sampling was done in Belgium (Flanders region).

**Fig. 1:**
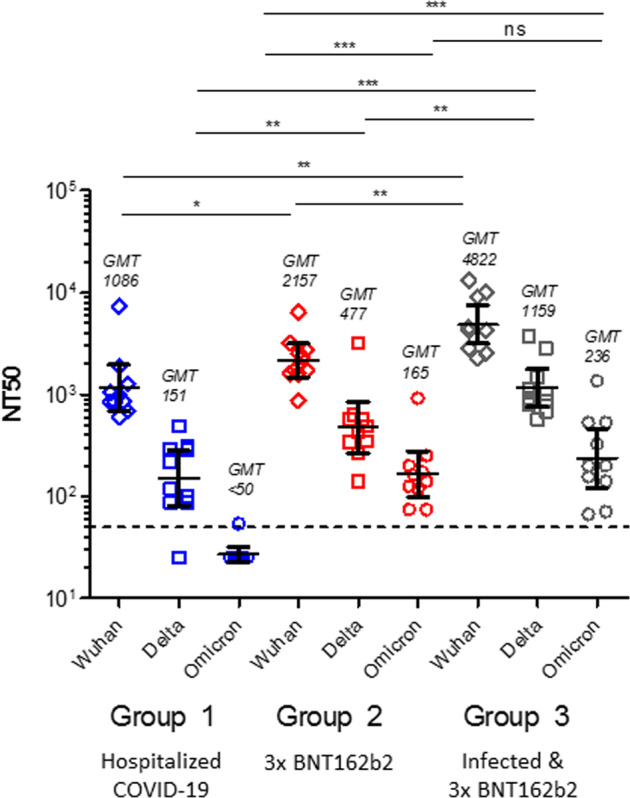
Neutralising antibody titre (NT50) against lineage B (Wuhan-Hu-1), Delta and Omicron variants. GMT (Geometric mean titer) with 95% CI, *P* values Mann–Whitney test: ***(< 0.001), **(< 0.01) for the three groups of samples analysed. The dotted line represents the assay cut-off (NT_50_ = 50). Additional statistical analysis can be found in Suppl. Table 1.

Individuals with immunity after infection but without vaccination (Group 1) have the lowest nAb responses regardless of the variant tested (Geometric mean neutralising titer 50% (NT50) (GMT)_Wuhan_ 1086 [corresponding to 1677 IU/ml], GMT_Delta_ 151 [234 IU/ml], GMT_Omicron_ < 50 [<77 IU/ml]) (Fig. 1). Infection-naïve individuals that received three doses of the BNT162b2 vaccine (Group 2) showed significantly higher nAb levels against lineage B (GMT_Wuhan_ 2157 [3331 IU/ml]), Delta (GMT_Delta_ 477 [737 IU/ml]) and Omicron (GMT_Omicron_ 165 [255 IU/ml]). Individuals with hybrid immunity (Group 3) showed the highest NT50 values against lineage B (GMT_Wuhan_ 4822 [7445 IU/ml]), Delta (GMT_Delta_ 1159 [1789 IU/ml]) and Omicron (GMT_Omicron_ 236 [363 IU/ml]) variants, but the nAb levels for Omicron were not statistically different between Groups 2 and 3.

We observed a significant reduction in NT50 across the three groups for the Delta variant (Group 1: 7.2-fold reduction; Group 2: 4.5-fold reduction; Group 3: 4.2-fold reduction in GMT) and a significantly larger reduction against the Omicron variant (Group 1: >22-fold reduction; Group 2: 13.1-fold reduction; Group 3: 20.5-fold reduction in GMT). This drop-in nAb titer is most pronounced for Group 1 (infection/unimmunized), but even for triple vaccinated subjects with (Group 3) or without (Group 2) previous infection, the NT50_Omicron_ is significantly lower than for the original lineage B and Delta strains (Suppl. Table 1). Our data confirm the earlier observation that three doses of the mRNA vaccine BNT162b2 result in high neutralising antibody titre for the three variants tested. Although hybrid immunity obtained through infection and subsequent vaccination results in significantly higher nAb levels against the original lineage B and Delta, the difference for Omicron is not statistically significant compared to the 3-dose BNT162b2 vaccine schedule in uninfected individuals (Suppl. Table 1). Of note, NT50_Omicron_ for Groups 2 and 3 are in the same range (non-significantly different) as NT50_Delta_ for Group 1.

In this study, we analysed only one critical component of immunity to SARS-CoV-2. Non-neutralising functions of antibodies and T-cell responses may be less affected by mutations of the Omicron variant and may thereby provide some compensation to immune escape^10^. A limitation of the study is that Group 1 is mainly composed of older-aged, hospitalised subjects with severe disease and multiple comorbidities in 50% of Group 1 subjects. It can thus not be excluded that immunosenescence or pre-existing comorbidities contribute to the lower antibody response observed in this group.

In conclusion, our findings confirm that three doses of the BNT162b2 vaccine confers neutralising antibody capacity against Omicron, and that vaccine-induced responses (Groups 2 and 3) outperform naturally-acquired immunity (Group 1). Hybrid immunity (Group 3) results in significantly higher nAb levels against the original lineage B and Delta, but not against Omicron. The observation that a two-dose schedule of BNT162b2 is not sufficient to neutralise Omicron^6,7,9^ warrants for rapid administration of a booster vaccine dose to counter infection and limit disease caused by this variant.

## Methods

### Virus isolation

An Omicron virus isolate was made from a nasopharyngeal (NP) swab collected from a patient with sequence-confirmed Omicron infection returning from a stay in the Republic of South Africa. An additional NP swab was collected 48 h after RT-qPCR diagnosis and 24 h after sequence confirmation of infection with the Omicron variant. The patient was infected 4.5 months after two doses of mRNA-1273 (Moderna) and presented with mild symptoms (cough, sore throat). The NP swap was collected on a universal transport medium (UTM) and transferred fresh to the BSL3 laboratory immediately after collection. Two times 200 µl of 1/2 diluted sample and 2 × 200 µl of 1/4 diluted sample (in Vero cell medium with 2% FBS) were added to an 80–100% confluent layer of Vero cells plated 1.5 h before in 24-well plates in Vero cell medium with 2% FBS (300.000 cells/well, plates were incubated at 37 °C and 7% CO_2_ before inoculation). Cells were spinoculated for 30 min at 2500 × *g* and 37 °C, then placed again for 10–15 min in the incubator, after which 800 µl of fresh Vero cell medium with 2% FBS was added. Plates were subsequently incubated at 37 °C and 7% CO_2_ and cytopathogenic effect (CPE) was scored daily by microscopy. On day 4 postinoculation, CPE was clearly visible in all inoculated wells and virus supernatant from all four wells was collected and pooled. The virus was further passaged a second time on Vero cells by adding 2 ml of the freshly collected virus suspension to 95–100% confluently grown Vero cells in a T175 culture flask and incubated for 2 h at 37 °C and 7% CO_2_. Next, the cells were washed with PBS, 35 ml of fresh Vero cell medium with 2% FBS was added and cells were incubated again at 37 °C and 7% CO_2_. On day 4 CPE was clearly visible and the virus was collected, centrifuged for 5 min at 3220 × *g* to remove debris, aliquoted and stored at −80 °C. Sequence confirmation regarding Omicron classification was obtained by sequencing the Spike gene on passage 1 of the viral isolate. Viral RNA was extracted using the QIAamp viral RNA mini kit (Qiagen) and the Spike coding sequence was RT-PCR amplified with primer sets COV2-800-39R (CAAAGGCACGCTAGTAGTCGTC), COV2-800-32L (GGGTGTTGCTATGCCTAATCTTTACA), COV2-800-38R (TGCAGTAGCGCGAACAAAATCT) and COV2-800-33L (TGCATGCAAATTACATATTTTGGAGGA), and subsequently sequenced with primers COV2-800-34L (GTTGGATGGAAAGTGAGTTCAGAGT), COV2-800-35L (TTCCGCATCATTTTCCACTTTTAAGT), COV2-800-36L (TGCACAGAAGTCCCTGTTGCTA), COV2-800-37L (TGCAGATGCTGGCTTCATCAAA), COV2-800-38L (CTTCCCTCAGTCAGCACCTCAT), COV2-800-32L (GGGTGTTGCTATGCCTAATCTTTACA), COV2-800-39R (CAAAGGCACGCTAGTAGTCGTC), COV2-800-33L (TGCATGCAAATTACATATTTTGGAGGA), and COV2-800-38R (TGCAGTAGCGCGAACAAAATCT) (adapted from^11^). Using BLAST+, the obtained Spike sequence was found to be identical to a recently published SARS-CoV-2 B.1.1.529 BA1 sequence from Belgium (GenBank accession number UFO69279.1).

### Neutralising antibody testing

SARS-CoV-2 neutralising antibodies were quantified by incubating serial dilutions of heat-inactivated serum (1/50–1/25,600 in EMEM supplemented with 2 mM l-glutamine, 100 U/ml–100 μg/ml of Penicillin–Streptomycin and 2% foetal bovine serum) during 1 h (37 °C, 7% CO_2_) with 3xTCID100 of wild type (WT) lineage B (2019-nCoV-Italy-INMI1, reference 008 V-03893), 83DJ-1 (B.1.617.2, Delta) or VLD20211207 (B.1.1.529, Omicron). One hundred µl of sample-virus mixtures and virus/cell controls were added to 100 µl of Vero cells (18,000 cells/well) in a 96-well plate and incubated for five days (37 °C, 7% CO_2_). The CPE caused by viral growth was scored microscopically. The Reed–Muench method was used to calculate the nAb titer that reduced the number of infected wells by 50% (NT50), which was used as a proxy for the nAb concentration in the sample^12,13^. In accordance with WHO guidance, an internal reference standard is included in each nAb assay run. This internal standard was calibrated against the International Standard 21/234 (NIBSC) and NT50 values were recalculated to IU/ml for each variant. The Mann–Whitney test was used to compare nAb titre between virus variants and groups. Data analysis was done using GraphPad Prism v5.03.

### Reporting Summary

Further information on research design is available in the Nature Research Reporting Summary linked to this article.

## Supplementary information

REPORTING SUMMARY

Suppl Table 1

## Data Availability

The datasets generated during and/or analysed during the current study are available from the corresponding author on reasonable request.
