# Supplementary material for: Three doses of BNT162b2 vaccine confer neutralising antibody capacity against the SARS-CoV-2 Omicron variant
Source: NPJ Vaccines. 2022 Mar 8;7:35. doi: 10.1038/s41541-022-00459-z (PMC8904765; doi:10.1038/s41541-022-00459-z)
Supplement: Supplementary file 1 — REPORTING SUMMARY [file 41541_2022_459_MOESM1_ESM.pdf]

nature portfolio

Corresponding author(s): Kevin K. Ariën

Last updated by author(s): Feb 14, 2022

Reporting Summary

Nature Portfolio wishes to improve the reproducibility of the work that we publish. This form provides structure for consistency and transparency in reporting. For further information on Nature Portfolio policies, see our Editorial Policies and the Editorial Policy Checklist.

Statistics

For all statistical analyses, confirm that the following items are present in the figure legend, table legend, main text, or Methods section.

n/a	Confirmed	
☐	☒	The exact sample size (n) for each experimental group/condition, given as a discrete number and unit of measurement
☐	☒	A statement on whether measurements were taken from distinct samples or whether the same sample was measured repeatedly
☐	☒	The statistical test(s) used AND whether they are one- or two-sided<br>*Only common tests should be described solely by name; describe more complex techniques in the Methods section.*
☒	☐	A description of all covariates tested
☒	☐	A description of any assumptions or corrections, such as tests of normality and adjustment for multiple comparisons
☐	☒	A full description of the statistical parameters including central tendency (e.g. means) or other basic estimates (e.g. regression coefficient) AND variation (e.g. standard deviation) or associated estimates of uncertainty (e.g. confidence intervals)
☒	☐	For null hypothesis testing, the test statistic (e.g. F, t, r) with confidence intervals, effect sizes, degrees of freedom and P value noted<br>*Give P values as exact values whenever suitable.*
☒	☐	For Bayesian analysis, information on the choice of priors and Markov chain Monte Carlo settings
☒	☐	For hierarchical and complex designs, identification of the appropriate level for tests and full reporting of outcomes
☒	☐	Estimates of effect sizes (e.g. Cohen's d, Pearson's r), indicating how they were calculated

Our web collection on statistics for biologists contains articles on many of the points above.

Software and code

Policy information about availability of computer code

Data collection	Data was collected in a validated Microsoft Excel worksheet
Data analysis	GraphPad Prism v5.03

For manuscripts utilizing custom algorithms or software that are central to the research but not yet described in published literature, software must be made available to editors and reviewers. We strongly encourage code deposition in a community repository (e.g. GitHub). See the Nature Portfolio guidelines for submitting code & software for further information.

Data

Policy information about availability of data

All manuscripts must include a data availability statement. This statement should provide the following information, where applicable:

- Accession codes, unique identifiers, or web links for publicly available datasets
- A description of any restrictions on data availability
- For clinical datasets or third party data, please ensure that the statement adheres to our policy

The data sets generated during and/or analysed during the current study are available from the corresponding author on reasonable request.

Field-specific reporting

Please select the one below that is the best fit for your research. If you are not sure, read the appropriate sections before making your selection.

☒ Life sciences ☐ Behavioural & social sciences ☐ Ecological, evolutionary & environmental sciences

For a reference copy of the document with all sections, see nature.com/documents/nr-reporting-summary-flat.pdf

Life sciences study design

All studies must disclose on these points even when the disclosure is negative.

Sample size	10 representative samples were selected based on clinical characteristics, no sample size calculation was done.
Data exclusions	No data was excluded from the analysis.
Replication	Six-fold replicates within the same assay were tested for each sample. Each series of experiments contained appropriate negative and positive controls, as well as an Internal Standard that was calibrated against the International Standard 21/234 (Working reagent for anti-SARS-CoV-2 immunoglobulin).
Randomization	Samples were assigned to groups based on clinical data and clinical diagnostic data.
Blinding	The investigators were not blinded to group allocation. The investigators were blinded to any patient identifiers.

Reporting for specific materials, systems and methods

We require information from authors about some types of materials, experimental systems and methods used in many studies. Here, indicate whether each material, system or method listed is relevant to your study. If you are not sure if a list item applies to your research, read the appropriate section before selecting a response.

Materials & experimental systems

n/a	Involved in the study	
☒	☐	Antibodies
☐	☒	Eukaryotic cell lines
☒	☐	Palaeontology and archaeology
☒	☐	Animals and other organisms
☐	☒	Human research participants
☐	☒	Clinical data
☒	☐	Dual use research of concern

Methods

n/a	Involved in the study	
☒	☐	ChIP-seq
☒	☐	Flow cytometry
☒	☐	MRI-based neuroimaging

Eukaryotic cell lines

Policy information about cell lines

Cell line source(s)	Vero cells obtained through ATCC (CCL-81).
Authentication	Authentication provided through ATCC.
Mycoplasma contamination	Vero cells tested negative for mycoplasma contamination.
Commonly misidentified lines (See ICLAC register)	not applicable.

Human research participants

Policy information about studies involving human research participants

Population characteristics	roup 1 sera (n=10) were obtained from COVID-19 patients hospitalized with severe infection, requiring ICU and ventilation. Half of these patients (5/10) had multiple comorbidities. All patients were infected between 24 Feb 2020 and 27 March 2020 when Wuhan D614G was the only variant circulating and when vaccines were not yet available. Samples tested were collected with a median time after onset of symptoms of 25 days [range 13-46]. Group 2 sera (n=10) were obtained from individuals without a documented previous SARS-CoV-2 infection and 28 days after third dose of BNT162b2. All individuals were vaccinated with a 21-day interval between dose 1 and 2 and received third dose 7 months (median 211 days [207-219]) after dose 2. Group 3 sera (n=10) were obtained from individuals with hybrid immunity, i.e. individuals who have had a previous infection with Wuhan D614G between 24 March 2020 and 11 June 2020, followed by three doses of the BNT162b2

	vaccine. Dose 1 and dose 2 were given 21-days apart and the third dose was administered with a median time interval of 8 months (median 261 days [218-290]) after dose 2. Samples tested for group 3 were collected with a median time after third vaccine dose of 14 days [range 10-82]. All participants were of European origin, with a mean age of 71 years [range 53-84; 40% females], 51 years [range 28-88, 70% females] and 67 years [range 22-95, 50% females] for groups 1, 2 and 3, respectively. Sampling was done in Belgium (Flanders region).
Recruitment	Group 1 samples are left-over samples from hospitalized patients with severe COVID-19. Group 2 and group 3 samples were collected from the prospective cohort study PICOV (Clinicaltrials.gov NCT04527614).
Ethics oversight	The study was approved by the Ethics Committee of Hôpital Erasme, Brussels, Belgium (reference B4062020000134), by the Federal Agency for Medicines and Health Products (2021-000401-24) and is registered on ClinicalTrials.gov (NCT04527614).

Note that full information on the approval of the study protocol must also be provided in the manuscript.

Clinical data

Policy information about clinical studies

All manuscripts should comply with the ICMJE guidelines for publication of clinical research and a completed CONSORT checklist must be included with all submissions.

Clinical trial registration	Clinicaltrials.gov NCT04527614
Study protocol	https://clinicaltrials.gov/ct2/show/study/NCT04527614
Data collection	not applicable for this study
Outcomes	not applicable for this study
