# Supplementary material for: Three doses of BNT162b2 vaccine confer neutralising antibody capacity against the SARS-CoV-2 Omicron variant
Source: NPJ Vaccines. 2022 Mar 8;7:35. doi: 10.1038/s41541-022-00459-z (PMC8904765; doi:10.1038/s41541-022-00459-z)
Supplement: Supplementary file 2 — Suppl Table 1 [file 41541_2022_459_MOESM2_ESM.pdf]

Suppl. Table 1: Statistical analysis nAb NT50 data intra-group and inter-group

		**group 1**			**group 2**			**group 3**		
		Wuhan	*delta*	*omicron*	*Wuhan*	*delta*	*omicron*	*Wuhan*	*delta*	*omicron*
**group 1**	*Wuhan*		***	***	*	**	***	**	ns	**
	delta			***	***	**	ns	***	***	ns
	omicron				***	***	***	***	***	***
**group 2**	*Wuhan*					**	***	**	*	***
	delta						**	***	**	ns
	omicron							***	***	ns
**group 3**	*Wuhan*								***	***
	delta									**
	omicron									

P values Mann-Whitney test

***(<0.001)

**(<0.01)

*(<0.05)

ns (not significant)

intra-group

inter-group
